# Insulin‐Like Growth Factor Axis and Survival in Metastatic Hormone‐Sensitive Prostate Cancer: Post‐Hoc Analysis From the ENZAMET Phase 3 Trial

**DOI:** 10.1002/pros.70245

**Published:** 2026-09-27

**Authors:** Michael A. Cilento, Hui‐Ming Lin, Nicole Yeung, Rachel M.N. Kim, Neil Portman, Martin R. Stockler, Andrew J. Martin, Vinod Subhash, Sonia Yip, Lisa M. Butler, Ian C. Marschner, Peter J. Meikle, Arun A. Azad, Ian D. Davis, Lisa G. Horvath, Christopher J. Sweeney

**Affiliations:** ^1^ South Australian Immunogenomics Cancer Institute Adelaide University Adelaide Australia; ^2^ Department of Medical Oncology Royal Adelaide Hospital Adelaide Australia; ^3^ Australia and New Zealand Urogenital and Prostate Cancer Trials Group (ANZUP) Sydney Australia; ^4^ Garvan Institute of Medical Research Sydney Australia; ^5^ School of Clinical Medicine University of New South Wales Sydney Australia; ^6^ NHMRC Clinical Trials Centre University of Sydney Camperdown Australia; ^7^ University of Sydney Camperdown Australia; ^8^ Department of Medical Oncology Chris O'Brien Lifehouse Sydney Australia; ^9^ The University of Queensland Brisbane Australia; ^10^ Baker Heart and Diabetes Institute Melbourne Australia; ^11^ Department of Cardiovascular Research Translation and Implementation La Trobe University Melbourne Australia; ^12^ Department of Medical Oncology Peter MacCallum Cancer Centre Melbourne Australia; ^13^ Sir Peter MacCallum Department of Oncology University of Melbourne Melbourne Australia; ^14^ Monash University Melbourne Australia; ^15^ Eastern Health Melbourne Australia

**Keywords:** biomarkers, chemotherapy, insulin‐like growth factor, prostate cancer

## Abstract

**Background:**

A higher ratio of IGF‐1:IGF binding protein 1 (IGFBP1) was associated with improved prognosis in the CHAARTED trial of docetaxel in participants with metastatic hormone‐sensitive prostate cancer (mHSPC). The ENZAMET trial demonstrated the survival benefit of adding enzalutamide to androgen deprivation therapy (ADT) in mHSPC, and included participants who also received docetaxel as standard of care. The aim of this study was to validate the association between levels of circulating IGF‐1 and IGFBP1 and survival of ENZAMET participants.

**Methods:**

IGF‐1 and IGFBP1 levels were measured in baseline plasma from 845 ENZAMET participants, and associations with clinical progression‐free survival (cPFS) and overall survival (OS) were assessed as a post‐hoc analysis.

**Results:**

A high baseline plasma ratio of IGF‐1:IGFBP1 (upper two tertiles) was associated with improved OS (HR 0.75, p 0.008) and improved cPFS (HR 0.82, p 0.034) compared to the lowest tertile. IGF‐1:IGFBP1 was not predictive of enzalutamide or docetaxel benefit (no significant interaction with treatment arm or docetaxel use). In participants in the enzalutamide arm, IGF‐1:IGFBP1 was an independent predictor for OS and cPFS in bivariable analysis with docetaxel use. The rate of high volume disease was similar in patients with lower IGF‐1:IGFBP1 ratio and those with high ratio (~50%).

**Conclusions:**

This analysis demonstrated a consistent association of improved prognosis with high IGF‐1:IGFBP1 in mHSPC, but did not identify which participants benefited from docetaxel when treated with enzalutamide.

## 1 Introduction

Metastatic hormone‐sensitive prostate cancer (mHSPC) guidelines recommend the use of androgen deprivation therapy (ADT) in combination with an androgen receptor pathway inhibitor (ARPI) [1, 2, 3]. The overall survival benefit from the use of the ARPI, enzalutamide, in combination with ADT over ADT alone was demonstrated by the ENZAMET [4] and ARCHES [5] phase 3 trials. Patients with high‐volume disease on conventional imaging derived the most OS benefit with docetaxel chemotherapy according to the earlier CHAARTED trial [6], which was subsequently confirmed by the STOPCaP meta‐analysis [7] including the GETUG15 [8] and STAMPEDE‐trials [9]. The addition of docetaxel to ADT + ARPI was shown to be consistently beneficial compared to ADT + docetaxel when evaluated in patient populations enriched for synchronous high‐volume mHSPC in the ARASENS [7], PEACE‐1 [10, 11], and ENZAMET phase 3 trials [12].

There is currently no reported prospective clinical trial comparing ADT + ARPI doublet therapy versus ADT + ARPI + docetaxel triplet therapy. Therefore, a question remains regarding the utility of adding docetaxel, beyond a contemporary ADT + ARPI doublet. Participants in ENZAMET were allowed to receive concurrent standard‐of‐care docetaxel at the clinician's discretion. Most participants also consented to collection of blood samples for translational research, allowing an opportunity to investigate potential circulating biomarkers of docetaxel's benefit.

Androgen receptor (AR) signaling is the dominant driver of prostate cancer biology and has complex interactions with the insulin‐like growth factor (IGF) axis [13, 14]. The IGF axis is a signaling network involving three ligands (IGF‐1, IGF‐2, insulin), their receptors (IGF1R, IGF2R, insulin receptor), and at least six IGF‐binding proteins (IGFBP1 to IGFBP6). The IGF‐binding proteins modulate the interaction of the ligands with their receptors [15], and cross‐reactivity between ligands and receptors exists. Experimental evidence has demonstrated the oncogenic role of IGF‐1 in the growth of prostate cancer cells [16] and in transgenic mice [17]. Observational data from international consortia has also supported the role of IGF‐1 in the development of prostate cancer including development of prostate cancer with aggressive features [18]. However, the clinical monoclonal IGF‐1R antibody cixutumumab did not increase undetectable PSA rates in mHSPC when added to ADT [19].

Higher circulating serum levels of IGFBP1 in people with mHSPC commencing ADT has been linked to a shorter time to development of castration‐resistant prostate cancer (CRPC) and shorter OS [20]. In previous research, there was a stepwise decrease in survival across tertiles of IGFBP1, with the highest tertile associated with the poorest outcomes [20]. This previous work led to the derivation and evaluation of the IGF‐1:IGFBP1 ratio in approximately 560 participants of the CHAARTED trial, where a higher serum IGF‐1:IGFBP1 ratio at baseline and after 6 months was associated with improved OS in both the ADT group and ADT + docetaxel group [21].

In this study we sought to validate the CHAARTED findings by assessing the performance of circulating IGF‐1 and IGFBP1 as prognostic biomarkers, and as predictive biomarkers of docetaxel benefit in triplet therapy in a large mHSPC cohort, by post‐hoc analysis of the ENZAMET trial.

## 2 Patients and Methods

### 2.1 Study Design and Participants

ENZAMET (ANZUP 1304; NCT02446405) is an international randomized phase 3 trial of ADT + enzalutamide versus ADT + conventional non‐steroidal antiandrogen (NSAA), with or without concurrent docetaxel, involving 1125 men with mHSPC [12]. Details of the eligibility criteria, study procedures, blinding, and power analysis were reported previously [12]. The trial protocol was independently reviewed and approved by the ethics committees of all the participating institutions, including the collection of biospecimens for future research. The trial was conducted in accordance with Good Clinical Practice guidelines and the Declaration of Helsinki. Participants were randomly allocated to treatment arms, with stratification for volume of disease, planned use of concurrent docetaxel, planned use of bone antiresorptive therapy, comorbidities, and study site, resulting in 562 in the control group (ADT + NSAA) versus 563 in the enzalutamide group (ADT + Enza). The trial met its primary endpoint confirming the addition of enzalutamide improves OS (HR 0.70, 95% CI 0.58–0.84; *p* < 0.0001), with an updated 8‐year landmark survival of 50% in those receiving enzalutamide compared to 40% in those receiving NSAA [22].

The biomarker study cohort for this analysis included baseline plasma samples from 845 participants of ENZAMET (427 from the control arm and 418 from the enzalutamide arm), all of whom provided consent to collection, storage, and use of biospecimens for research (Figure 1A). Patients without baseline plasma samples available were excluded. The study was approved by the local ethics committee.

**Figure 1:**
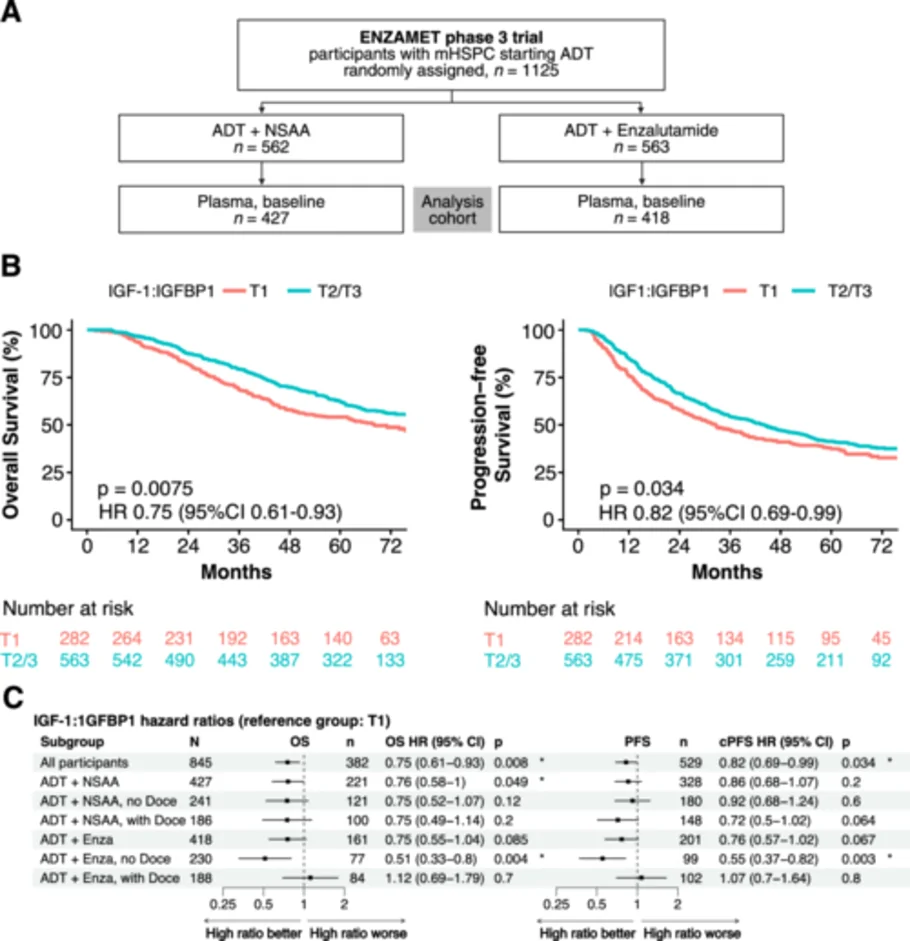
Overview of the biomarker study cohort (A), overall survival and clinical progression free survival of IGF‐1:IGFBP1 lower tertile (T1) versus upper two tertiles (T2/T3) (B), progression free survival and overall survival according to subgroups (C). ADT, androgen deprivation therapy; Doce, docetaxel; Enza, enzalutamide; IGF‐1, insulin‐like growth factor‐1; IGFBP1, insulin‐like growth factor‐binding protein 1; NSAA, non‐steroidal antiandrogen; OS, overall survival; PFS, progression‐free survival. [Color figure can be viewed at wileyonlinelibrary.com]

### 2.2 Quantitation of IGF Proteins

IGF‐1 and IGFBP1 were measured in baseline plasma samples using MILLIPLEX assays (Human IGF‐1, II Magnetic Bead Panel; Human IGFBP Magnetic Bead Panel) by Merck (Darmstadt, Germany) according to manufacturer's instructions. The fluorescence output from the assays was quantitated with the MAGPIX system (Luminex Corporation, Austin, TX). The concentration of the analytes in the samples on each plate was calculated from the median fluorescence intensity and calibration standards on the same plate, using the instrument's analysis software (Xponent v4.2.1324, Luminex Corporation). To correct batch differences, the concentrations were normalized by global median centering, with the first plate of each assay as the reference for all other plates. Normalized concentrations with values that were outside the range of the calibration standards were replaced with the concentration of the nearest calibration standard.

### 2.3 Statistical Analysis

The IGF‐1:IGFBP1 ratio was calculated by dividing the normalized IGF‐1 value (ng/mL) with the normalized IGFBP1 value (ng/mL). The ratio was transformed into log2 scale. The selection of an appropriate threshold of IGF‐1:IGFBP1 to describe the biomarker levels as high versus low, was first determined by assessing IGF‐1:IGFBP1 as a binary variable with various cutpoints in Cox regression analysis for associations with clinical outcomes (OS and cPFS). The binary cutpoints assessed included the lower tertile, median, and upper tertile. The cutpoint for defining the lower tertile of the log2 IGF‐1:IGFBP1 ratio is ≤ 3.032. All statistical analysis were performed with R using the R packages survival, survminer, gtsummary and tidyverse. No new code was generated in this study.

## 3 Results

### 3.1 Patient Characteristics

In total, 427 of 562 (76%) participants from the control arm and 418 of 563 (74%) from the enzalutamide arm were included in the analysis. The high number of samples in this analysis resulted in a biomarker study population that was well‐matched for baseline characteristics (Table 1), in keeping with the overall population of the randomized ENZAMET trial. The baseline levels of the IGF markers also did not differ between treatment arms (Table 1).

**Table 1:** Baseline characteristics of biomarker cohort.

Characteristic	Conventional NSAA *N* = 427^a^	Enzalutamide *N* = 418^a^
Volume of disease		
Low	199 (47%)	195 (47%)
High	228 (53%)	223 (53%)
Concurrent docetaxel	186 (44%)	188 (45%)
ACE‐27 score		
0–1	317 (74%)	315 (75%)
2–3	110 (26%)	103 (25%)
Anti‐resorptive therapy	31 (7.3%)	32 (7.7%)
Region		
ANZ	274 (64%)	290 (69%)
Ireland/UK	43 (10%)	45 (11%)
North America	110 (26%)	83 (20%)
Gleason grade		
≤ 7	122 (33%)	111 (31%)
8–10	243 (67%)	251 (69%)
Unknown	62	56
ECOG performance score		
0	313 (73%)	301 (72%)
1–2	114 (27%)	117 (28%)
Age		
< 70	237 (56%)	222 (53%)
≥ 70	190 (44%)	196 (47%)
Visceral metastases	52 (12%)	48 (11%)
Timing of metastases		
Metachronous/unknown	154 (36%)	169 (40%)
Synchronous	273 (64%)	249 (60%)
IGF‐1 (ng/ml)	47 (30, 72)	49 (27, 71)
IGFBP1 (ng/ml)	2.7 (1.4, 5.0)	2.7 (1.3, 5.1)
IGF‐1:IGFBP1 ratio	16 (5, 43)	16 (5, 42)

^a^ n (%) or median (Q1, Q3).

### 3.2 Association of Baseline IGF‐1:IGFBP1, IGF‐1, and IGFBP1 With Survival

The association of baseline IGF‐1:IGFBP with survival was largely consistent across the three different binary cutpoints of lower tertile, median or upper tertile (Supporting Information Figure S1). Therefore, in order to harmonize our results with previously published work, binary IGF‐1:IGFBP1 ratio with the lower tertile as its cut‐point (3.032) was taken forward for further analysis [21], that is, participants in the upper two tertiles (T2/T3) of IGF‐1:IGFBP1 were considered to have a high ratio (> 3.032), whereas those in the lower tertile (T1) were considered to have a low ratio (≤ 3.032).

A high IGF‐1:IGFBP1 was significantly associated with improved OS (HR 0.75, 95% CI 0.61–0.93; p 0.008) and improved cPFS (HR 0.82, 95%CI 0.69‐0.99; p 0.034) in the overall population compared to the low ratio (Figure 1). Unlike the ratio, baseline IGF‐1 or IGFBP1 as continuous or binary variables were generally not significantly associated with outcomes (Supporting Information Figures S2 and S3).

### 3.3 Effect of Enzalutamide Versus Control According to IGF‐1:IGFBP1

The overall survival benefit of enzalutamide versus control was similar in both subgroups of participants according to IGF‐1:IGFBP1 ratio. Participants with low ratio (T1) of IGF‐1:IGFBP1 had an OS HR 0.66 (95% CI: 0.48–0.93) and those with high ratio (T2/T3) had an OS HR 0.65 (95% CI: 0.50–0.84). However, none of the IGF markers assessed, including high‐ versus low‐ratio, were predictive of enzalutamide response as there was no significant interaction with treatment arm (interaction *p*‐value > 0.9 for ratio).

### 3.4 Prognostic Impact of IGF‐1:IGFBP1 Ratio, Enzalutamide Arm

In the enzalutamide arm, analysis of the subgroup of participants who received the ADT + enzalutamide doublet (without docetaxel) showed that a high ratio was associated with better OS and cPFS (*p* < 0.05) (Figure 2).

**Figure 2:**
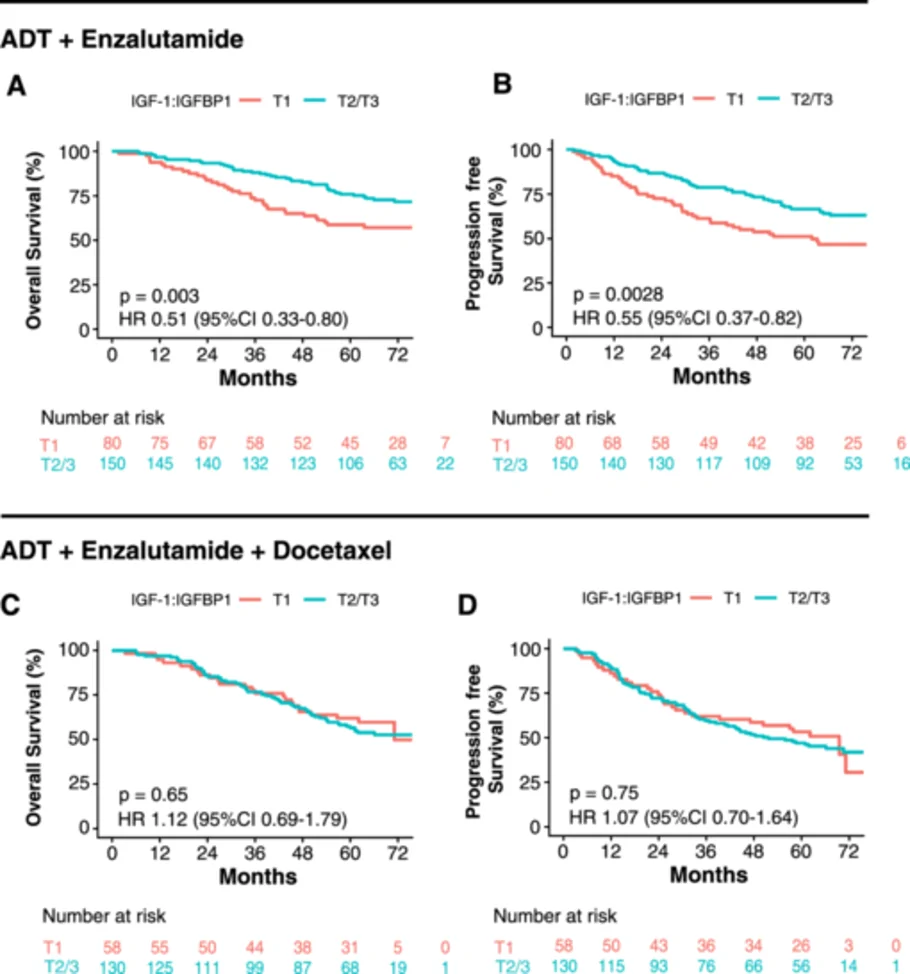
Survival of participants randomized to the enzalutamide arm separated by low‐ versus high‐ratio, participants who received ADT + enzalutamide doublet OS (A) and PFS (B) and participants who received ADT + enzalutamide + docetaxel triplet OS (C) and PFS (D). ADT, androgen deprivation therapy; IGF‐1, insulin‐like growth factor‐1; IGFBP1, insulin‐like growth factor‐binding protein 1; NSAA, non‐steroidal antiandrogen; OS, overall survival; PFS, progression‐free survival. [Color figure can be viewed at wileyonlinelibrary.com]

Some participants in the enzalutamide arm also received docetaxel as their standard‐of‐care therapy at their clinician's discretion. In those randomized to receive enzalutamide, participants with a low ratio of IGF‐1:IGFBP1 (T1) had a 5‐year OS of 60% (95% CI: 52–69) and 5‐year cPFS of 52% (95% CI: 44–61) and did not derive any benefit from the addition of docetaxel compared to those who did not receive docetaxel (*p* > 0.8)(Figure 3B). Participants with a high IGF‐1:IGFBP1 ratio (T2/3) and randomized to enzalutamide had a 5‐year OS of 67% (95% CI: 62%–73%) and cPFS of 58% (95% CI: 52%–64%). Docetaxel use was not randomized in the trial, but rather selected for individuals based on clinician discretion. It has previously been reported that participants most frequently selected for concurrent docetaxel had poor prognostic disease [4]. Noting this imbalance, within the T2/T3 subgroup, those who received docetaxel had worse OS (HR 1.99, 95% CI 1.34–2.97; *p* < 0.001) and cPFS (HR 1.89, 95%CI 1.33–2.68; *p* < 0.001) compared to those who did not receive docetaxel (Figure 3C), again reflecting the known enrichment of poor‐prognosis disease in this subgroup. The corresponding 5‐year OS and cPFS for those without docetaxel were 76% (95% CI: 69–83) and 67% (95% CI: 59–75) and for those receiving docetaxel were 57% (95% CI: 49–66) and 47% (95% CI: 39–57).

**Figure 3:**
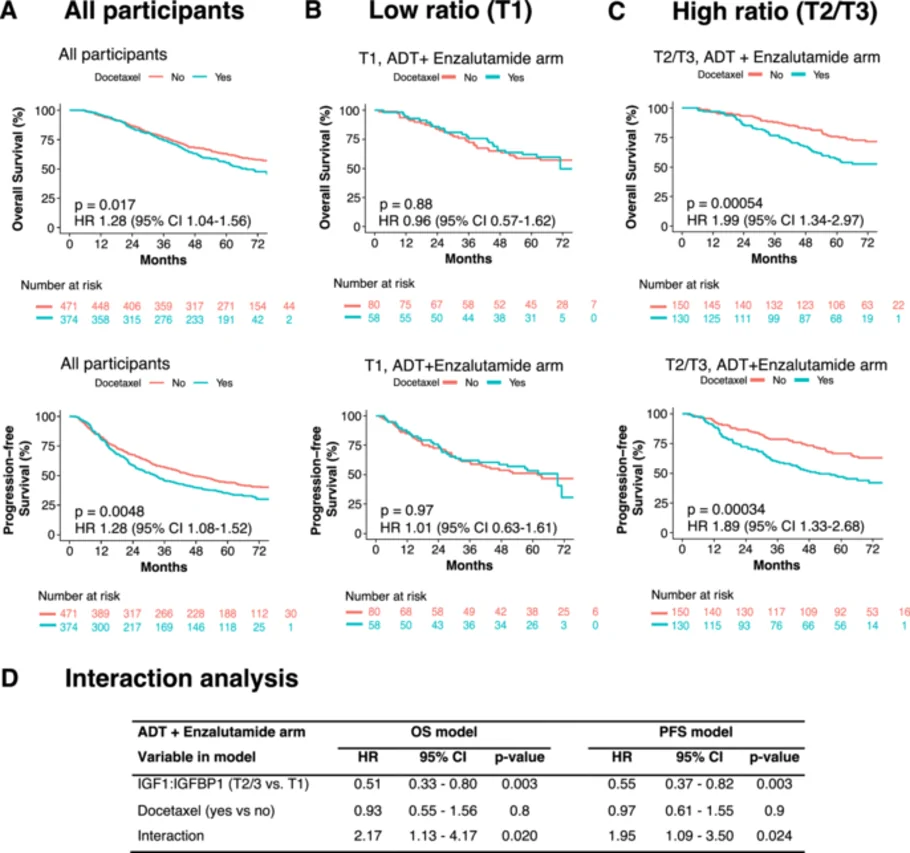
Overall survival and progression free survival according to docetaxel use. Survival curves of all participants (A), participants with low ratio (B) and high ratio (C), interaction analysis (D). ADT, androgen deprivation therapy; IGF‐1, insulin‐like growth factor‐1; IGFBP1, insulin‐like growth factor‐binding protein 1; NSAA, non‐steroidal antiandrogen; OS, overall survival; PFS, progression‐free survival; T1, tertile 1; T2/T3, tertile 2 and tertile 3. [Color figure can be viewed at wileyonlinelibrary.com]

In an exploratory subgroup of participants enriched for the highest risk, based on the combination of both T1 ratio and high volume disease, exploratory findings suggest adding docetaxel to ADT + enzalutamide may benefit these participants more. In the enzalutamide arm those with T1 and high‐volume who had docetaxel added had OS HR 0.62 (95% CI 0.34–1.18), which was a unique finding when compared to the T2/T3 high‐volume subgroup (OS HR 1.55, 95% CI 0.93–2.58), the T1 low‐volume subgroup (OS HR 1.66, 95% CI 0.61–4.51), and the T2/T3 low‐volume subgroup (OS HR 1.30, 95% CI 0.59–2.85). Importantly these findings are exploratory only, given the low patient numbers per subgroup (Supporting Information Figure S4).

### 3.5 High‐Volume Disease and Synchronous Disease

Among participants with high IGF‐1:IGFBP1 (T2/3) in the enzalutamide arm, there was a higher percentage of high‐volume disease (72% vs 35%) and synchronous disease (73% vs 44%) in those receiving docetaxel compared to those not receiving docetaxel (Table 2). Notably, in participants with low IGF‐1:IGFBP1 (T1), there was no significant difference in use of docetaxel or associated high volume or synchronous status.

**Table 2:** Volume of disease and timing of metastases in the enzalutamide arm by docetaxel use and IGF1:IGFBP1 ratio.

ADT + Enza	High IGF1:IGFBP1 (T2/T3) docetaxel		*p* value^a^	Low IGF1:IGFBP1 (T1) docetaxel		*p* value^a^
	No	Yes		No	Yes	
Volume of disease			< 0.001			0.14
Low	98 (65%)	37 (28%)		39 (49%)	21 (36%)	
High	52 (35%)	93 (72%)		41 (51%)	37 (64%)	
Timing of metastases			< 0.001			0.3
Metachronous/unknown	84 (56%)	35 (27%)		32 (40%)	18 (31%)	
Synchronous	66 (44%)	95 (73%)		48 (60%)	40 (69%)	

^a^ Pearson's Chi‐squared test.

### 3.6 Prognostic Impact of Disease Volume or Diabetes Status

Multivariable analysis (enzalutamide arm) of IGF‐1:IGFBP1 with docetaxel, volume of disease (high vs. low), and timing of metastases (synchronous vs. metachronous), demonstrated that high‐volume disease is the only independent predictor for OS and cPFS after accounting for the other three factors, with 2.6 times (95% CI 1.83–3.75; *p* < 0.001) higher risk of death than patients with low‐volume disease (Figure 4A). There was no difference seen in the range of IGF markers according to volume of disease (Figure 4B). The rate of high volume disease by IGF‐1:IGFBP1 ratio also did not differ (*p* = 0.2, Figure 4C). The percentage of participants with low IGF‐1:IGFBP1 ratio (T1, *n* = 282) who had high volume disease was 56%, whereas the percentage of those with high IGF‐1:IGFBP1 ratio (T2/T3, *n* = 563) who had high volume disease was 52%.

**Figure 4:**
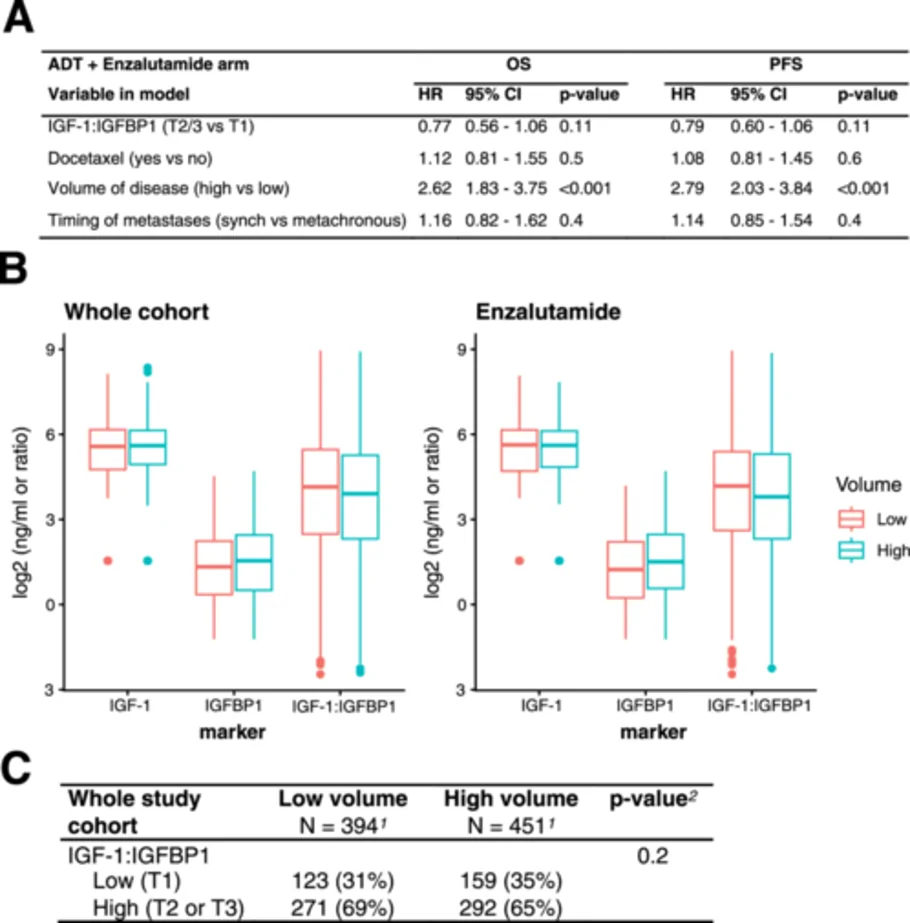
Multivariable Cox regression of IGF‐1:IGFBP1, docetaxel, volume of disease and timing of metastases for the enzalutamide arm, with OS and PFS (A); Box plots of IGF markers (log2) between high volume and low volume disease in whole cohort and enzalutamide subgroup (B), frequency of IGF‐1 subgroups by volume of disease (C). ADT, androgen deprivation therapy; IGF‐1, insulin‐like growth factor‐1; IGFBP1, insulin‐like growth factor‐binding protein 1; OS, overall survival; PFS, progression‐free survival; T1, tertile 1; T2/T3, tertile 2 and tertile 3. [Color figure can be viewed at wileyonlinelibrary.com]

There was also no difference seen in the range of IGF markers in participants with diabetes compared to those without diabetes (Supporting Information Figure S5). The association of IGF‐1:IGFBP1 with OS and cPFS was independent of diabetes status (data not shown, hazard ratios and *p*‐values for IGF‐1:IGFBP1 similar to those in Figure 1).

## 4 Discussion

A higher ratio of IGF‐1:IGFBP1 at baseline is associated with improved OS and cPFS in this analysis of 845 participants with mHSPC from the ENZAMET trial. Our study represents one of the largest analysis of the IGF‐axis in a randomized phase 3 clinical trial to date, with 75% of all ENZAMET participants included. It is also the first study to report relationships between the IGF‐axis and outcomes in patients treated with ADT + ARPI. This work validates earlier evidence from clinical cohorts of mHSPC demonstrating the prognostic impact of circulating markers of the IGF axis.

The importance of the relative levels of IGF‐1 and IGFBP1 was previously shown in a retrospective cohort analysis of 122 patients commencing ADT within the preceding 3 months, which assessed time to CRPC and OS [20]. In this cohort there was an association between baseline IGFBP1 levels and time to CRPC (median time to CRPC in low, middle, and top IGFBP1 tertiles was 20.7, 18.1, and 12.4 months, respectively) indicating a better prognosis for individuals with the lowest IGFBP1 levels [20]. Analysis of the IGF‐1:IGFBP1 ratio showed the same favorable prognosis in those patients with a high ratio (reflecting low IGFBP1 levels, or high IGF1 levels). Similarly, serial assessments from CHAARTED confirmed the association with improved OS with a higher IGF‐1:IGFBP1 ratio [21].

These two previous studies were performed with serum samples whereas our work was conducted on plasma specimens. Serum levels often do not correlate well with plasma levels and concentrations can differ according to the analytical methods employed [23]. Despite these factors, our findings are consistent with those of the previous research, thus reliably demonstrating that higher IGF‐1:IGFBP1 is associated with a better prognosis regardless of whether serum or plasma is tested, and the type of platform used.

The underlying role of IGFs as growth‐promoting peptides occurs in a complex biological network, which is driven by the interaction with their receptors which is dependent on, and modulated by, their interaction with binding proteins [24]. IGF‐1 promotes tumor cell survival and proliferation by binding to its receptor, IGF‐1R, which results in phosphorylation of several cellular proteins, and downstream activation of important signaling pathways such as the PI3K‐ and MAPK‐pathways [25].

In the circulation, IGF‐1 is mostly bound to IGFBP1 which modulates its action and extends its half‐life. Higher levels of circulating IGF‐1 have been associated with increased risk of several cancers including prostate cancer, which may reflect intracellular signaling that promotes cancer cell survival [26]. Previous preclinical work has suggested binding proteins may have a role in sequestering IGF‐1 from its receptor resulting in apoptosis [27, 28, 29, 30]. In our study, the IGF‐1:IGFB1 ratio is a better prognostic indicator than IGF‐1 alone. The association of a high IGF‐1:IGFBP1 ratio (i.e. more IGF‐1 than IGFBP1 in circulation) with improved prognosis contradicts the expected association of high IGF‐1 with poorer prognosis. Similarly, pre‐clinical work has also suggested that IGF‐1 may play a role in docetaxel resistance [31], this too is at odds with our finding of the apparent benefit of docetaxel in patients with low IGF‐1:IGFBP1. Further research will therefore be required to better understand the mechanistic role of circulating versus tumor‐derived IGFs in this setting.

In the clinical setting there is a need for circulating biomarkers to help decide which people with mHSPC can benefit from adding docetaxel to ADT + ARPI. To explore this, we looked further into the results of participants assigned to the ADT + enzalutamide arm comparing those who received docetaxel to those who did not. Even after accounting for IGF‐1:IGFBP1, high‐volume disease remained the only independent prognostic indicator of OS and PFS. The levels of IGF‐1 and IGFBP1 were not associated with the volume of disease.

There was no difference in OS and PFS between participants who received docetaxel in addition to enzalutamide versus those who did not if they were in the lowest tertile of IGF‐1:IGFBP1. However, the use of docetaxel in enzalutamide‐treated participants with the higher tertiles of IGF‐1:IGFBP1 was associated with a worse OS. This is likely due to the poorer prognostic clinical factors that led to their physician recommending concurrent docetaxel. As such, our analysis of the predictive ability of IGF‐1:IGFBP1 for survival benefit from docetaxel is confounded by the volume of disease and synchronous timing of metastases. Thus, there is insufficient evidence to determine if IGF‐1:IGFBP1 can be used to choose who benefits from adding docetaxel to ADT + enzalutamide.

Limitations of this research include the retrospective nature of the analysis, the relatively small sample size when focusing on different subgroups, and that docetaxel use was not randomized. This study, although the largest of its kind, would also benefit from further exploration in clinical trials that are specifically designed to test the additional impact of docetaxel to an ADT + ARPI doublet, such as the ASPIRE phase 3 trial (NCT06931340) or the TRIPLE‐SWITCH phase 3 trial (NCT06592924).

## 5 Conclusion

A high IGF‐1:IGFBP1 ratio is a biological variable consistently associated with improved OS and PFS across multiple cohorts of people with mHSPC and implicates the IGF‐axis in the biology of prostate cancer and its potential for biomarker and drug development. However, this biological variable cannot be used to help identify individuals who benefit from addition of docetaxel.

## Ethics Statement

The study was approved by St Vincent Hospital's human research ethics (reference number 12/231).

## Conflicts of Interest

M.A.C. declares no competing interests.

H.M.L. and L.G.H. are inventors of a patent for a prognostic lipid biomarker of metastatic prostate cancer licensed by Chris O'Brien Lifehouse (PCT/AU2023/050849). LGH is a member of the International Trial Steering Committee for ENZAMET, Board member of ANZUP and Chair, ANZUP Prostate Subcommittee (unpaid); has received research funding paid to institution by Astellas and Bayer and has compensated advisory board roles for Amgen, Janssen, Astellas, MSD and Bayer.

L.M.B. is a member of the Scientific Advisory Committee and Board of ANZUP (unpaid).

C.J.S. declares being PI of CHAARTED, Study Co‐Chair ENZAMET, and member of the ANZUP Scientific Advisory Committee (unpaid); has received research funding paid to institution by Janssen (apalutamide), Astellas (enzalutamide), Pfizer (enzalutamide), Sanofi (docetaxel, cabazitaxel), Bayer (darolutamide) and had compensated advisory roles for Sanofi (docetaxel, cabazitaxel), Janssen (apalutamide), Astellas (enzalutamide), Bayer (darolutamide), Genentech/Roche (ipatasertib), Pfizer (enzalutamide), Lilly (abemaciclib), Hengrui, PointBiopharma (PNT2002); Novartis (LuPSMA) and holds patents for parthenolide as an anti‐cancer agent and parthenolide and analogs as a radiation protectant. Use of the drug combination of abiraterone and cabozantinib to treat prostate cancer with Dr. Philip Kantoff), Compositions and methods for screening and diagnosis of prostate cancer with KDM5D biomarker with co‐authors Drs Philip Kantoff, Gwo‐Shu Mary Lee and Kazumasa Komura (WO2017/117486 A1, owned by Dana‐Farber Cancer Institute) Compositions and methods for screening and identifying clinically aggressive prostate cancer with SNPs and ZFP36 co‐authored by Drs. Curtis Huttenhower; Travis Gerke; Christopher Sweeney; Lorelei Mucci; Gwo‐Shu Mary Lee; Daniela Bornigen stock ownership in Leuchemix and Parthera for developing dimethylaminoparthenolide formulations.

## Supporting information


**Figure S1:** HR of baseline IGF1:IGFBP1 ratio analysed as a binary variable defined by cutpoints of the lower tertile (3.032, a), median (4.003, b) or upper tertile (4.822, c), and as a log2 continuous variable (d), in univariable Cox regression for OS and clinical PFS.

**Figure S2:** HR of baseline IGF‐1 analysed as a binary variable defined by cutpoints of the lower tertile (5.155, a), median (5.597, b), or upper tertile (5.932, c), and as a continuous variable (d), in univariable Cox regression for OS and clinPFS. File 2.

**Figure S3:** HR of baseline IGFBP1 (log2 of ng/ml) analysed as a binary variable defined by cut‐points of the lower tertile (0.858, a), median (1.426, b), or upper tertile (2.054, c), and as a continuous variable (d), in univariable Cox regression for OS and clinPFS.

**Figure S4:** Overall survival and progression free survival in the enzalutamide arm according to docetaxel use. Participants with T1 and high volume disease (a), T1 and low volume disease (b), T2/T3 and high volume disease (c), T2/T3 and low volume disease (d).

**Figure S5:** Box plots of IGF markers (log2) between diabetes and no diabetes in whole cohort and enzalutamide subgroup.

## Data Availability

The data that support the findings of this study are available on request from the corresponding author. The data are not publicly available due to privacy or ethical restrictions.
