# Supplementary material for: Insulin‐Like Growth Factor Axis and Survival in Metastatic Hormone‐Sensitive Prostate Cancer: Post‐Hoc Analysis From the ENZAMET Phase 3 Trial
Source: Prostate. 2026 Sep 27;86(16):1671–80. doi: 10.1002/pros.70245 (PMC13768792; doi:10.1002/pros.70245)

Figure S5

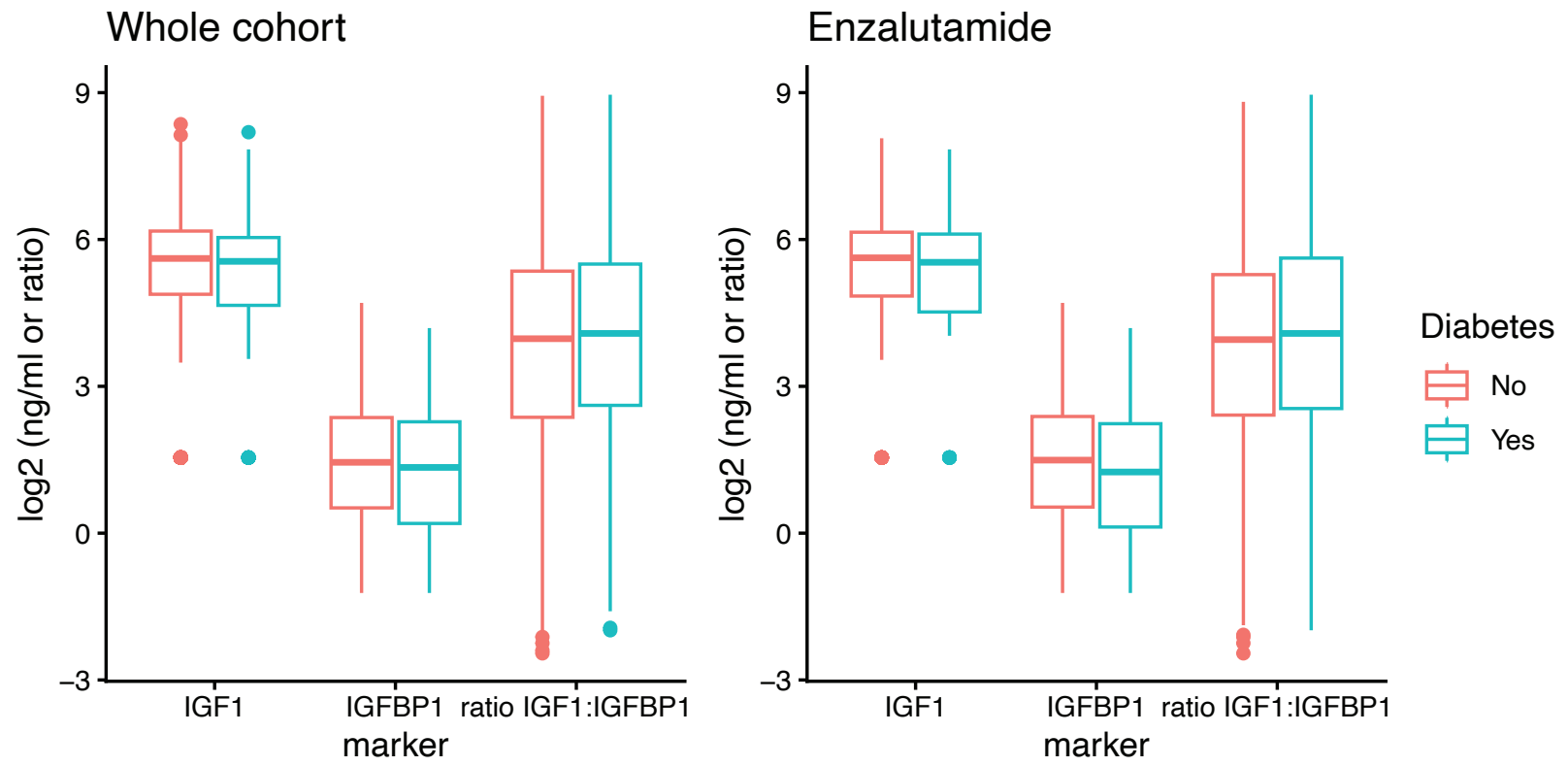

n, No	n, Diabetes	Whole cohort	Fold (95% CI)	P−value
680	164	IGF1	1.01 (0.83−1.23)	>0.9
680	164	IGFBP1	0.87 (0.74−1.03)	0.10
680	164	IGF1:IGFBP1	1.16 (0.89−1.51)	0.3

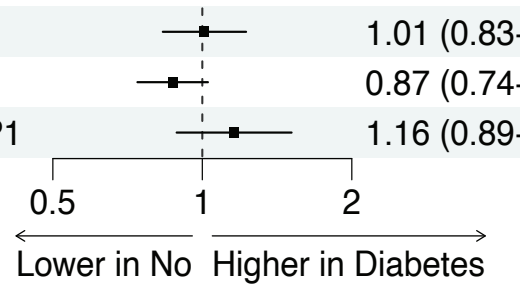

n, No	n, Diabetes	Enzalutamide	Fold (95% CI)	P−value
325	92	IGF1	0.9 (0.69−1.17)	0.4
325	92	IGFBP1	0.82 (0.65−1.03)	0.084
325	92	IGF1:IGFBP1	1.1 (0.76−1.59)	0.6

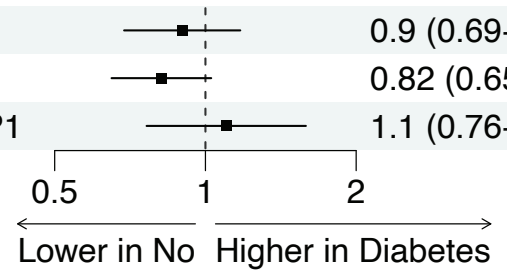

Supplement: Supplementary file 1 [file PROS-86-1671-s001.pdf]
