# Supplementary material for: Insulin‐Like Growth Factor Axis and Survival in Metastatic Hormone‐Sensitive Prostate Cancer: Post‐Hoc Analysis From the ENZAMET Phase 3 Trial
Source: Prostate. 2026 Sep 27;86(16):1671–80. doi: 10.1002/pros.70245 (PMC13768792; doi:10.1002/pros.70245)
Supplement: Supplementary file 2 [file PROS-86-1671-s002.pdf]

Figure S1

A

IGF1:IGFBP1 (tert1)	N	OS outcome	n	OS HR (95% CI)	p		cPFS outcome	n	cPFS HR (95% CI)	p	
All participants	845		382	0.75 (0.61–0.93)	0.008	*		529	0.82 (0.69–0.99)	0.034	*
ADT + NSAA	427		221	0.76 (0.58–1)	0.049	*		328	0.86 (0.68–1.07)	0.2	
ADT + NSAA, no DCX	241		121	0.75 (0.52–1.07)	0.12			180	0.92 (0.68–1.24)	0.6	
ADT + NSAA, with DCX	186		100	0.75 (0.49–1.14)	0.2			148	0.72 (0.5–1.02)	0.064	
ADT + Enza	418		161	0.75 (0.55–1.04)	0.085			201	0.76 (0.57–1.02)	0.067	
ADT + Enza, no DCX	230		77	0.51 (0.33–0.8)	0.004	*		99	0.55 (0.37–0.82)	0.003	*
ADT + Enza, with DCX	188		84	1.12 (0.69–1.79)	0.7			102	1.07 (0.7–1.64)	0.8	

0.25 0.5 1 2 — OS HR (95% CI) — High ratio better / High ratio worse

0.25 0.5 1 2 — cPFS HR (95% CI) — High ratio better / High ratio worse

B

IGF1:IGFBP1 (median)	N	OS outcome	n	OS HR (95% CI)	p		cPFS outcome	n	cPFS HR (95% CI)	p	
All participants	845		382	0.75 (0.62–0.92)	0.006	*		529	0.79 (0.66–0.94)	0.006	*
ADT + NSAA	427		221	0.81 (0.62–1.05)	0.11			328	0.88 (0.71–1.09)	0.3	
ADT + NSAA, no DCX	241		121	0.73 (0.51–1.04)	0.081			180	0.89 (0.66–1.19)	0.4	
ADT + NSAA, with DCX	186		100	0.91 (0.61–1.35)	0.6			148	0.85 (0.62–1.17)	0.3	
ADT + Enza	418		161	0.69 (0.5–0.94)	0.019	*		201	0.66 (0.5–0.87)	0.003	*
ADT + Enza, no DCX	230		77	0.51 (0.32–0.81)	0.004	*		99	0.51 (0.34–0.77)	0.001	*
ADT + Enza, with DCX	188		84	0.93 (0.6–1.42)	0.7			102	0.85 (0.57–1.25)	0.4	

0.25 0.5 1 2 — OS HR (95% CI) — High ratio better / High ratio worse

0.25 0.5 1 2 — cPFS HR (95% CI) — High ratio better / High ratio worse

C

IGF1:IGFBP1 (tert2)	N	OS outcome	n	OS HR (95% CI)	p		cPFS outcome	n	cPFS HR (95% CI)	p	
All participants	845		382	0.76 (0.61–0.95)	0.014	*		529	0.82 (0.68–0.99)	0.037	*
ADT + NSAA	427		221	0.78 (0.58–1.04)	0.093			328	0.92 (0.73–1.15)	0.5	
ADT + NSAA, no DCX	241		121	0.7 (0.46–1.06)	0.088			180	0.85 (0.61–1.17)	0.3	
ADT + NSAA, with DCX	186		100	0.86 (0.57–1.3)	0.5			148	0.97 (0.7–1.35)	0.9	
ADT + Enza	418		161	0.73 (0.52–1.03)	0.075			201	0.7 (0.52–0.95)	0.023	*
ADT + Enza, no DCX	230		77	0.7 (0.42–1.16)	0.2			99	0.64 (0.41–1.01)	0.057	
ADT + Enza, with DCX	188		84	0.74 (0.47–1.18)	0.2			102	0.72 (0.48–1.1)	0.13	

0.25 0.5 1 2 — OS HR (95% CI) — High ratio better / High ratio worse

0.25 0.5 1 2 — cPFS HR (95% CI) — High ratio better / High ratio worse

D

IGF1:IGFBP1 (log2)	N	OS outcome	n	OS HR (95% CI)	p		cPFS outcome	n	cPFS HR (95% CI)	p	
All participants	845		382	0.94 (0.9–0.98)	0.008	*		529	0.96 (0.92–0.99)	0.015	*
ADT + NSAA	427		221	0.95 (0.9–1.01)	0.081			328	0.97 (0.93–1.02)	0.3	
ADT + NSAA, no DCX	241		121	0.94 (0.87–1.02)	0.14			180	0.97 (0.91–1.04)	0.4	
ADT + NSAA, with DCX	186		100	0.95 (0.87–1.04)	0.3			148	0.96 (0.9–1.03)	0.3	
ADT + Enza	418		161	0.93 (0.88–1)	0.042	*		201	0.93 (0.87–0.98)	0.011	*
ADT + Enza, no DCX	230		77	0.88 (0.8–0.97)	0.007	*		99	0.89 (0.82–0.96)	0.004	*
ADT + Enza, with DCX	188		84	0.98 (0.9–1.08)	0.7			102	0.96 (0.88–1.05)	0.4	

0.5 1 2 — OS HR (95% CI) — High ratio better / High ratio worse

0.5 1 2 — cPFS HR (95% CI) — High ratio better / High ratio worse
