# Supplementary material for: Insulin‐Like Growth Factor Axis and Survival in Metastatic Hormone‐Sensitive Prostate Cancer: Post‐Hoc Analysis From the ENZAMET Phase 3 Trial
Source: Prostate. 2026 Sep 27;86(16):1671–80. doi: 10.1002/pros.70245 (PMC13768792; doi:10.1002/pros.70245)
Supplement: Supplementary file 3 [file PROS-86-1671-s003.pdf]

Figure S3

A

IGFBP1 (tert1)	N	n	OS HR (95% CI)	p	n	cPFS HR (95% CI)	p
All participants	845	382	1.13 (0.91–1.4)	0.3	529	1.14 (0.95–1.37)	0.2
ADT + NSAA	427	221	1.04 (0.78–1.39)	0.8	328	1.04 (0.82–1.32)	0.7
ADT + NSAA, no DCX	241	121	1.11 (0.75–1.64)	0.6	180	1.07 (0.78–1.46)	0.7
ADT + NSAA, with DCX	186	100	0.97 (0.64–1.48)	0.9	148	1.02 (0.72–1.44)	>0.9
ADT + Enza	418	161	1.19 (0.86–1.66)	0.3	201	1.18 (0.88–1.58)	0.3
ADT + Enza, no DCX	230	77	1.35 (0.83–2.18)	0.2	99	1.28 (0.84–1.95)	0.3
ADT + Enza, with DCX	188	84	1.04 (0.66–1.63)	0.9	102	1.07 (0.71–1.62)	0.7

OS outcome: OS HR (95% CI), axis 0.5, 1, 2, 4; ← High levels better | High levels worse →

cPFS outcome: cPFS HR (95% CI), axis 0.5, 1, 2, 4; ← High levels better | High levels worse →

B

IGFBP1 (median)	N	n	OS HR (95% CI)	p		n	cPFS HR (95% CI)	p	
All participants	845	382	1.26 (1.03–1.54)	0.024	*	529	1.18 (0.99–1.4)	0.061	
ADT + NSAA	427	221	1.19 (0.91–1.55)	0.2		328	1.1 (0.89–1.37)	0.4	
ADT + NSAA, no DCX	241	121	1.43 (1–2.04)	0.052		180	1.21 (0.9–1.62)	0.2	
ADT + NSAA, with DCX	186	100	0.93 (0.63–1.38)	0.7		148	0.97 (0.7–1.33)	0.8	
ADT + Enza	418	161	1.36 (1–1.86)	0.052		201	1.32 (1–1.74)	0.051	
ADT + Enza, no DCX	230	77	1.5 (0.96–2.36)	0.077		99	1.28 (0.86–1.9)	0.2	
ADT + Enza, with DCX	188	84	1.22 (0.79–1.87)	0.4		102	1.35 (0.91–1.99)	0.14	

OS outcome: OS HR (95% CI), axis 0.5, 1, 2, 4; ← High levels better | High levels worse →

cPFS outcome: cPFS HR (95% CI), axis 0.5, 1, 2, 4; ← High levels better | High levels worse →

C

IGFBP1 (tert2)	N	n	OS HR (95% CI)	p		n	cPFS HR (95% CI)	p	
All participants	845	382	1.29 (1.05–1.59)	0.017	*	529	1.2 (1–1.43)	0.045	*
ADT + NSAA	427	221	1.2 (0.91–1.57)	0.2		328	1.06 (0.85–1.34)	0.6	
ADT + NSAA, no DCX	241	121	1.38 (0.96–1.98)	0.086		180	1.12 (0.83–1.52)	0.5	
ADT + NSAA, with DCX	186	100	0.99 (0.66–1.5)	>0.9		148	0.99 (0.7–1.4)	>0.9	
ADT + Enza	418	161	1.38 (1–1.9)	0.048	*	201	1.38 (1.04–1.84)	0.028	*
ADT + Enza, no DCX	230	77	1.52 (0.96–2.4)	0.073		99	1.47 (0.98–2.2)	0.064	
ADT + Enza, with DCX	188	84	1.25 (0.8–1.96)	0.3		102	1.29 (0.86–1.93)	0.2	

OS outcome: OS HR (95% CI), axis 0.5, 1, 2, 4; ← High levels better | High levels worse →

cPFS outcome: cPFS HR (95% CI), axis 0.5, 1, 2, 4; ← High levels better | High levels worse →

D

IGFBP1 (log2)	N	n	OS HR (95% CI)	p		n	cPFS HR (95% CI)	p	
All participants	845	382	1.12 (1.04–1.21)	0.004	*	529	1.09 (1.02–1.16)	0.011	*
ADT + NSAA	427	221	1.1 (0.99–1.21)	0.084		328	1.05 (0.97–1.15)	0.2	
ADT + NSAA, no DCX	241	121	1.15 (1–1.33)	0.052		180	1.06 (0.94–1.19)	0.3	
ADT + NSAA, with DCX	186	100	1.04 (0.9–1.2)	0.6		148	1.05 (0.93–1.19)	0.4	
ADT + Enza	418	161	1.14 (1.02–1.27)	0.025	*	201	1.12 (1.02–1.24)	0.022	*
ADT + Enza, no DCX	230	77	1.23 (1.04–1.44)	0.014	*	99	1.16 (1.01–1.34)	0.036	*
ADT + Enza, with DCX	188	84	1.05 (0.9–1.23)	0.5		102	1.08 (0.94–1.25)	0.3	

OS outcome: OS HR (95% CI), axis 0.5, 1, 2; ← High levels better | High levels worse →

cPFS outcome: cPFS HR (95% CI), axis 0.5, 1, 2; ← High levels better | High levels worse →
