# Supplementary material for: Insulin‐Like Growth Factor Axis and Survival in Metastatic Hormone‐Sensitive Prostate Cancer: Post‐Hoc Analysis From the ENZAMET Phase 3 Trial
Source: Prostate. 2026 Sep 27;86(16):1671–80. doi: 10.1002/pros.70245 (PMC13768792; doi:10.1002/pros.70245)
Supplement: Supplementary file 4 [file PROS-86-1671-s004.pdf]

**Figure S4**

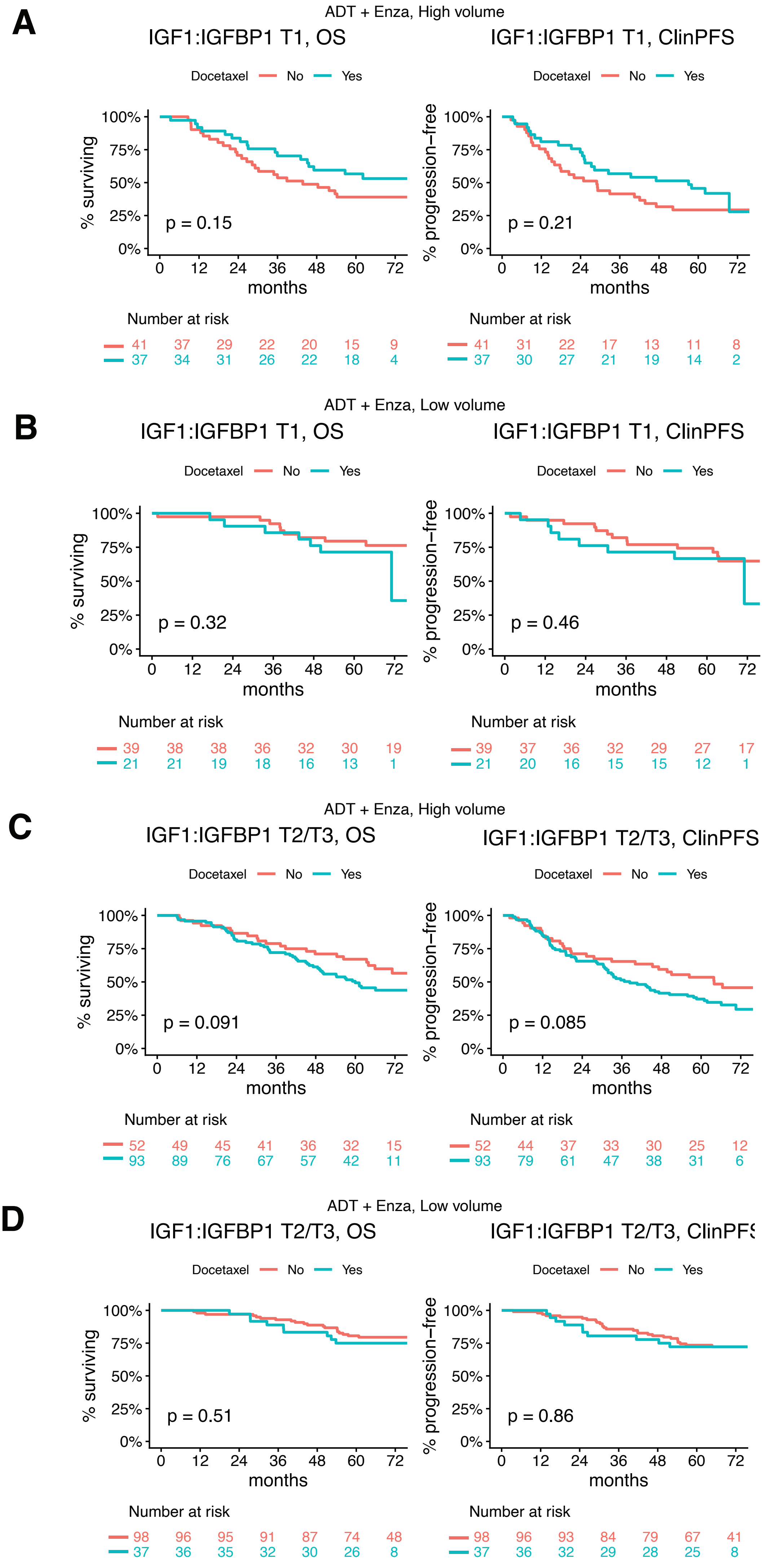
A
ADT + Enza, High volume
IGF1:IGFBP1 T1, OS
IGF1:IGFBP1 T1, ClinPFS
Docetaxel No Yes
% surviving
% progression−free
100%
75%
50%
25%
0%
p = 0.15
p = 0.21
0 12 24 36 48 60 72
months
Number at risk
41 37 29 22 20 15 9
37 34 31 26 22 18 4
41 31 22 17 13 11 8
37 30 27 21 19 14 2
B
ADT + Enza, Low volume
IGF1:IGFBP1 T1, OS
IGF1:IGFBP1 T1, ClinPFS
p = 0.32
p = 0.46
39 38 38 36 32 30 19
21 21 19 18 16 13 1
39 37 36 32 29 27 17
21 20 16 15 15 12 1
C
ADT + Enza, High volume
IGF1:IGFBP1 T2/T3, OS
IGF1:IGFBP1 T2/T3, ClinPFS
p = 0.091
p = 0.085
52 49 45 41 36 32 15
93 89 76 67 57 42 11
52 44 37 33 30 25 12
93 79 61 47 38 31 6
D
ADT + Enza, Low volume
IGF1:IGFBP1 T2/T3, OS
IGF1:IGFBP1 T2/T3, ClinPFS
p = 0.51
p = 0.86
98 96 95 91 87 74 48
37 36 35 32 30 26 8
98 96 93 84 79 67 41
37 36 32 29 28 25 8
