# Supplementary material for: Insulin‐Like Growth Factor Axis and Survival in Metastatic Hormone‐Sensitive Prostate Cancer: Post‐Hoc Analysis From the ENZAMET Phase 3 Trial
Source: Prostate. 2026 Sep 27;86(16):1671–80. doi: 10.1002/pros.70245 (PMC13768792; doi:10.1002/pros.70245)

Figure S2

A

IGF1 (tert1)	N	n	OS HR (95% CI)	p	n	cPFS HR (95% CI)	p
All participants	845	382	0.89 (0.72–1.1)	0.3	529	0.91 (0.76–1.08)	0.3
ADT + NSAA	427	221	0.89 (0.67–1.18)	0.4	328	0.99 (0.79–1.25)	>0.9
ADT + NSAA, no DCX	241	121	0.86 (0.6–1.24)	0.4	180	0.96 (0.71–1.3)	0.8
ADT + NSAA, with DCX	186	100	0.9 (0.58–1.4)	0.6	148	1 (0.7–1.44)	>0.9
ADT + Enza	418	161	0.88 (0.64–1.22)	0.4	201	0.78 (0.59–1.04)	0.10
ADT + Enza, no DCX	230	77	0.7 (0.44–1.1)	0.12	99	0.67 (0.45–1)	0.050
ADT + Enza, with DCX	188	84	1.1 (0.69–1.75)	0.7	102	0.91 (0.6–1.37)	0.6

OS outcome

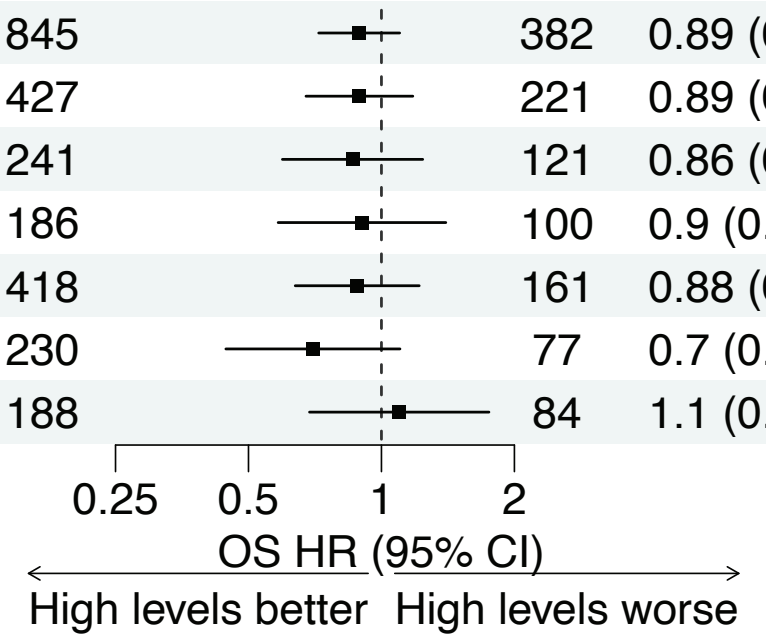

cPFS outcome

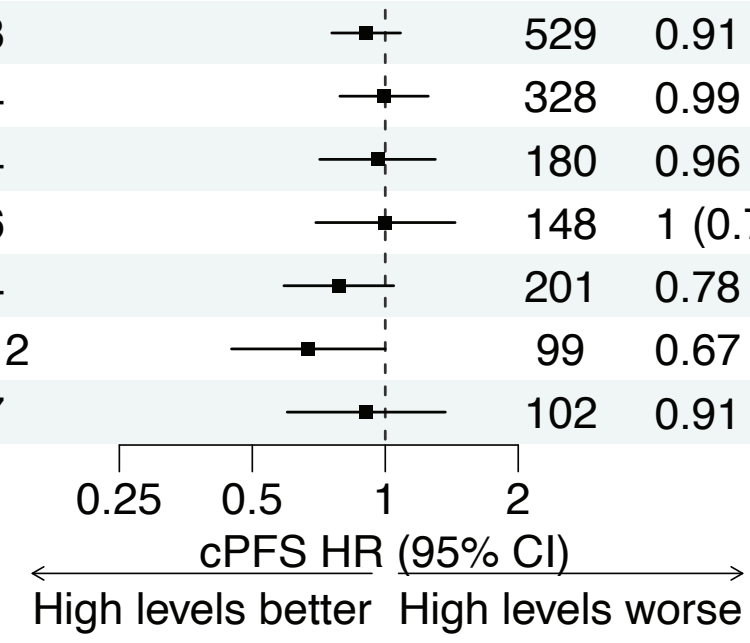

B

IGF1 (median)	N	n	OS HR (95% CI)	p	n	cPFS HR (95% CI)	p	
All participants	845	382	0.86 (0.7–1.05)	0.13	529	0.86 (0.72–1.02)	0.082	
ADT + NSAA	427	221	0.91 (0.7–1.18)	0.5	328	0.98 (0.79–1.21)	0.8	
ADT + NSAA, no DCX	241	121	0.93 (0.64–1.33)	0.7	180	0.98 (0.73–1.31)	0.9	
ADT + NSAA, with DCX	186	100	0.84 (0.57–1.25)	0.4	148	0.91 (0.65–1.25)	0.5	
ADT + Enza	418	161	0.82 (0.6–1.12)	0.2	201	0.77 (0.58–1.01)	0.063	
ADT + Enza, no DCX	230	77	0.64 (0.41–1)	0.052	99	0.66 (0.44–0.98)	0.042	*
ADT + Enza, with DCX	188	84	1.03 (0.67–1.58)	>0.9	102	0.87 (0.59–1.29)	0.5	

OS outcome

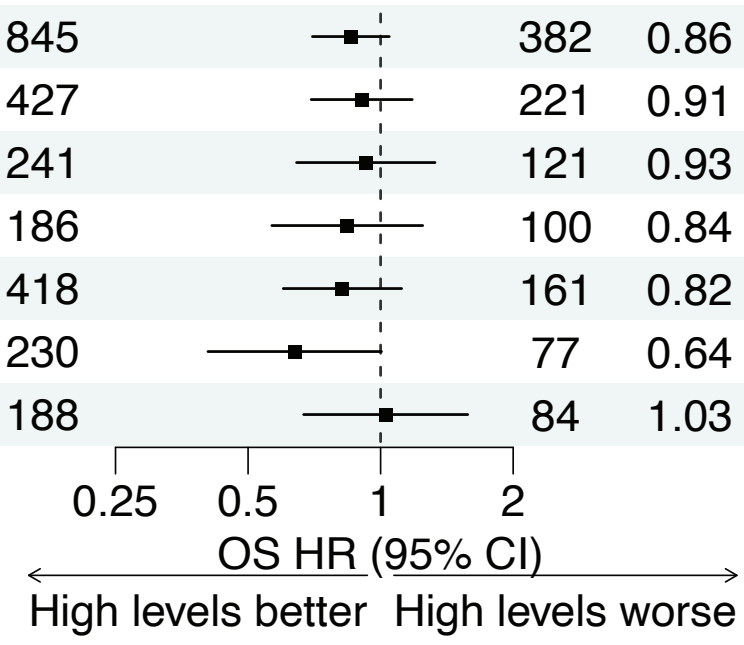

cPFS outcome

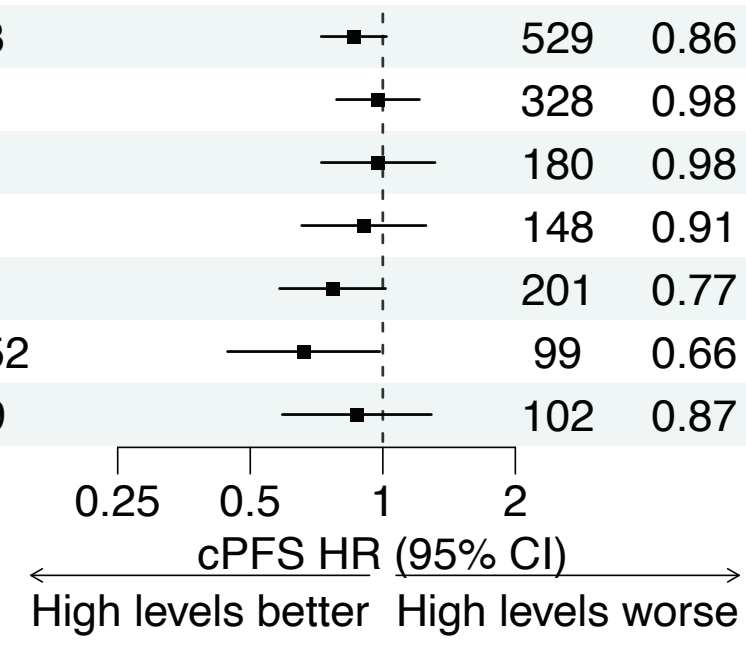

C

IGF1 (tert2)	N	n	OS HR (95% CI)	p	n	cPFS HR (95% CI)	p
All participants	845	382	0.9 (0.72–1.11)	0.3	529	0.89 (0.74–1.07)	0.2
ADT + NSAA	427	221	0.91 (0.69–1.21)	0.5	328	0.95 (0.76–1.19)	0.7
ADT + NSAA, no DCX	241	121	1.11 (0.75–1.65)	0.6	180	1.04 (0.75–1.43)	0.8
ADT + NSAA, with DCX	186	100	0.7 (0.47–1.05)	0.083	148	0.81 (0.58–1.12)	0.2
ADT + Enza	418	161	0.85 (0.61–1.2)	0.4	201	0.76 (0.56–1.03)	0.077
ADT + Enza, no DCX	230	77	0.8 (0.48–1.32)	0.4	99	0.71 (0.45–1.12)	0.14
ADT + Enza, with DCX	188	84	0.88 (0.56–1.4)	0.6	102	0.76 (0.5–1.16)	0.2

OS outcome

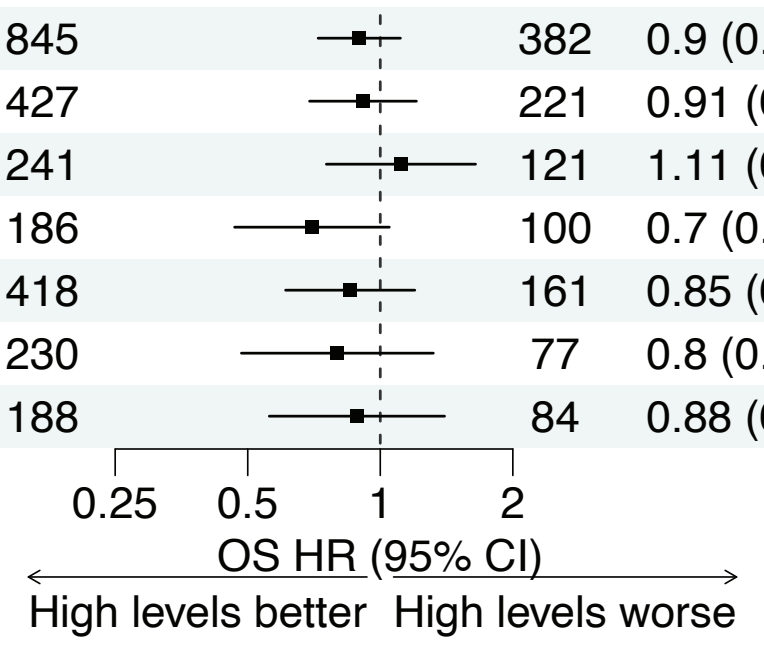

cPFS outcome

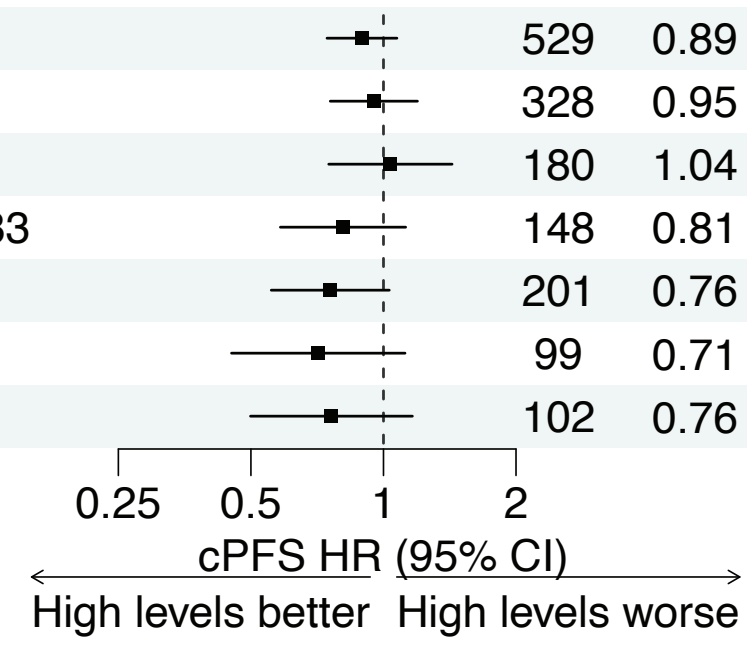

D

IGF1 (log2)	N	n	OS HR (95% CI)	p	n	cPFS HR (95% CI)	p	
All participants	845	382	0.96 (0.91–1.02)	0.2	529	0.97 (0.92–1.02)	0.2	
ADT + NSAA	427	221	0.96 (0.89–1.04)	0.3	328	0.98 (0.92–1.05)	0.6	
ADT + NSAA, no DCX	241	121	0.97 (0.87–1.08)	0.6	180	0.99 (0.9–1.08)	0.8	
ADT + NSAA, with DCX	186	100	0.93 (0.83–1.05)	0.3	148	0.96 (0.87–1.06)	0.4	
ADT + Enza	418	161	0.96 (0.88–1.05)	0.4	201	0.94 (0.87–1.02)	0.13	
ADT + Enza, no DCX	230	77	0.91 (0.81–1.02)	0.12	99	0.9 (0.81–0.99)	0.037	*
ADT + Enza, with DCX	188	84	1.01 (0.88–1.15)	>0.9	102	0.98 (0.87–1.11)	0.8	

OS outcome

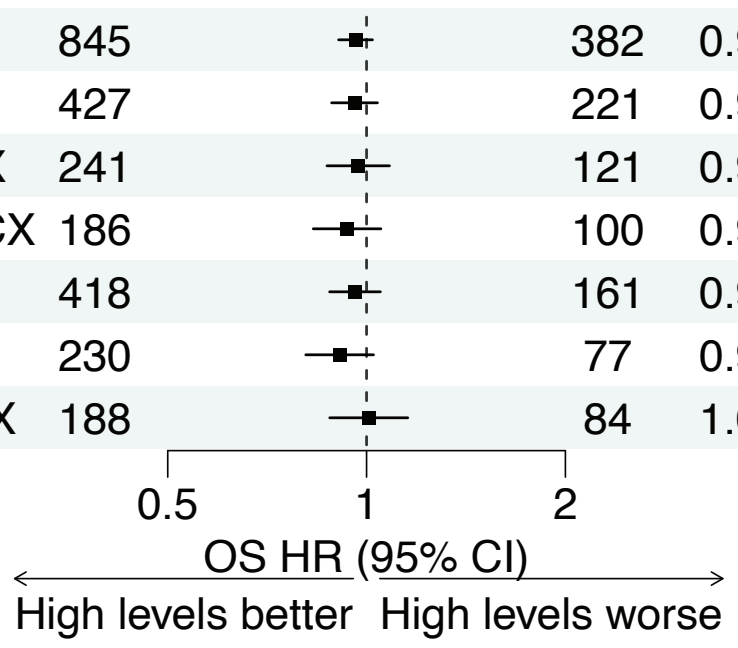

cPFS outcome

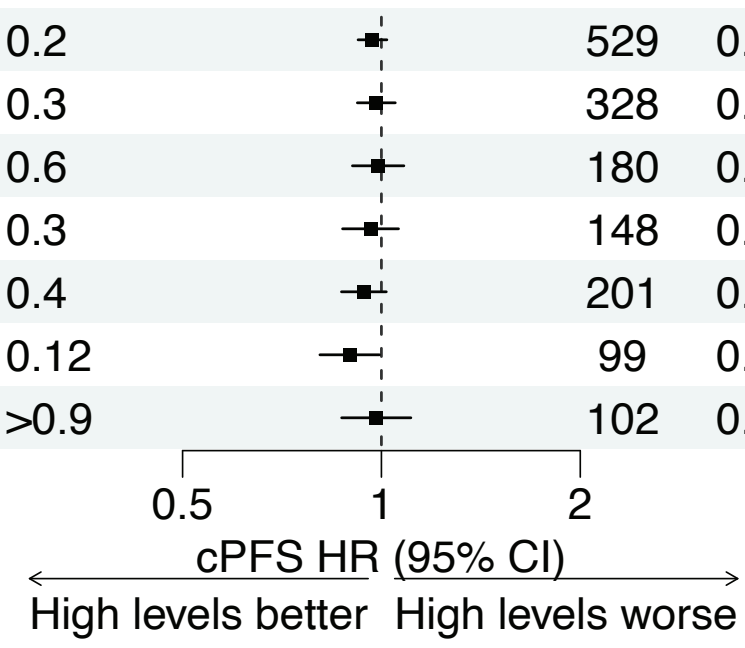

Supplement: Supplementary file 5 [file PROS-86-1671-s005.pdf]
